# First-Pass Metabolism of Polyphenols from Selected Berries: A High-Throughput Bioanalytical Approach

**DOI:** 10.3390/antiox9040311

**Published:** 2020-04-13

**Authors:** Francisco J. Olivas-Aguirre, Sandra Mendoza, Emilio Alvarez-Parrilla, Gustavo A. Gonzalez-Aguilar, Monica A. Villegas-Ochoa, Jael T.J. Quintero-Vargas, Abraham Wall-Medrano

**Affiliations:** 1 Departamento de Ciencias de la Salud, Universidad de Sonora (Campus Cajeme), Blvd Bordo Nuevo s/n, Ejido Providencia, Cd, Obregón 85199, Mexico; jael.quintero@unison.mx; 2 Departamento de Investigación y Posgrado en Alimentos (PROPAC), Facultad de Química, Universidad Autónoma de Querétaro, Cerro de las Campanas s/n, Santiago de Querétaro 76010, Mexico; smendoza@uaq.mx; 3 Instituto de Ciencias Biomédicas, Universidad Autónoma de Ciudad Juárez, Anillo Envolvente del PRONAF y Estocolmo s/n, Ciudad Juárez 32310, Mexico; ealvarez@uacj.mx; 4 Centro de Investigación en Alimentación y Desarrollo, A.C. (CIAD), Carretera a Ejido La Victoria, Km. 0.6, Hermosillo 83304, Mexico; gustavo@ciad.mx (G.A.G.-A.); mvillegas@ciad.mx (M.A.V.-O.)

**Keywords:** anthocyanins, berries, polyphenols, bioaccesibility, differential pulse voltammetry, first-pass metabolism, HPLC-ESI-QTOF-MS, apparent permeability

## Abstract

Small berries are rich in polyphenols whose first-pass metabolism may alter their ultimate physiological effects. The antioxidant capacity and polyphenol profile of three freeze-dried berries (blackberry, raspberry, Red Globe grape) were measured and their apparent permeability (Papp) and first-pass biotransformation were tracked with an ex vivo bioanalytical system [everted gut sac (rat) + three detection methods: spectrophotometry, HPLC-ESI-QTOF-MS, differential pulse voltammetry (DPV)]. Total polyphenol (ratio 0.07-0.14-1.0) and molecular diversity (anthocyanins > flavan-3-ols), antioxidant capacity (DPPH, FRAP), anodic current *maxima* and Papp (efflux> uptake) were in the following order: blackberry > raspberry > Red Globe grape. Epicatechin, pelargonidin & cyanin (all), callistephin (raspberry/blackberry), catechin (grape), cyanidin glycosides (blackberry) and their derived metabolites [quinic acid, epicatechin, cyanidin/malvidin glucosides, and chlorogenic/caffeic acids] were fruit-specific and concentration-dependent. Time-trend DPV kinetic data revealed concurrent epithelial permeability & biotransformation processes. Regular permeability and high-biotransformation of berry polyphenols suggest fruit-specific health effects apparently at the intestinal level.

## 1. Introduction

Regular consumption of small berries has been associated with several health benefits. Epidemiological studies and controlled clinical trials indicate that their acute-chronic consumption exerts synergistic and independent effects on lowering several pathophysiological markers including hyperglycemia, hyperinsulinemia, dyslipidemia, pro-inflammatory cytokines, hypertensive factors and oxidative stressors [1]. In fact, there is also an inverse association between berry consumption and many risk factors for cardiovascular disease and type-2 diabetes [2]; most, if not all, of these health benefits, are related to the amount and phytochemical diversity present in each berry, from which those with antioxidant activity (e.g., polyphenols) have been the most studied [3]. However, the heterogeneity in physiological response after their intake can hinder their beneficial effects in specific subpopulations [1,4]. 

To exert their physiological effects, berry polyphenols must be present in sufficient amounts in raw or prepared foods, be both bioaccessible (the fraction released from the food matrix during gastrointestinal digestion) and bioavailable (the fraction that reaches systemic circulation as the parent compound or a metabolite). The bioaccessibility of polyphenols is closely related to their physicochemical structure, the food matrix that contains them and the presence of anti-nutritional factors that could interfere with their release ability and intestinal absorption [5]. In this sense, the bioaccessibility of polyphenols from berry fruits is higher compared to other fruits, due to a concomitant effect between their natural higher level [6] and their low content of non-digestible carbohydrates and protein, both associated with an efficient gastrointestinal (GI) delivery that enhances their bioavailability [2,7].

On the other hand, the absorption, pharmacokinetics and systemic metabolism of polyphenols and their and biotransformation by the GI microbiota, have been extensively studied in the last decade [2,5]; however, the first-pass metabolism (a.k.a. pre-systemic metabolism) of bioaccessible polyphenols also modifies their ultimate health effects [1]. Polyphenols present in plant foods are commonly biotransformed (e.g., conjugation, de-glycation) before and during their pre-systemic passage, a phenomenon that involves several brush-border enzymes and a tightly-regulated influx/efflux interchange that sustain their cellular homeostasis [8,9]. These events are not normally considered when evaluating the pharmacokinetics of polyphenols, partially due to the absence of high sensitivity methods [10,11] to record such metabolic changes. 

The evaluation of bioaccessible and bioavailable fractions of polyphenols has been proposed recently as a “quality” parameter in berry breeding programs. However, studies reporting the extent to which bioaccessible polyphenols are bio-transformed during their first-pass metabolism and colonic fermentation are still very scarce [2,9]. In this study, we used a high-throughput ex vivo bioanalytical system [everted gut sac (rat) + three detection methods: spectrophotometry, HPLC-ESI-QTOF-MS, differential pulse voltammetry (DPV)] to evaluate the apparent permeability (*P_app_*) and enteral biotransformation of polyphenols from three berry fruits with graded levels of polyphenols (blackberry > raspberry > Red Globe grape); to the best of our knowledge, this ex vivo bioanalytical approach is reported for the first time.

## 2. Materials and Methods 

### 2.1. Chemicals and Standards

Pure (≥ 93%) chemical standards were purchased from Cayman Chemicals (Ann Arbor, MI, USA). 2,2-Diphenyl-1-picrylhydrazyl (DPPH), 2,2’-azobis(2-amidinopropane) dihydrochloride (AAPH), 6-hydroxy-2,5,7,8-tetramethylchroman-2-carboxylic acid (Trolox), fluorescein, Folin–Ciocalteu (FC) phenol reagent, ACS-grade salts & acids and all enzymes and chemicals used for the *in vitro* digestion and *ex vivo* apparent permeability assays, were purchased from Sigma-Aldrich Fluka (St. Louis, MO, USA). Analytical and HPLC-MS grade solvents were obtained from JT-Baker (Avantor Performance Materials S.A. de C.V., Ecatepec de Morelos, Estado de Mexico, Mexico); sodium pentobarbital (Pisabental^®^) was acquired from PISA Agropecuaria (Guadalajara, Jalisco, Mexico).

### 2.2. Samples and Extracts

Fully ripe Red Globe grape (*Vitis vinifera* L., 18° Brix, pH 4), raspberry (*Rubus idaeus L,* 10° Brix, pH 3) and blackberry (*Rubus* spp., 10° Brix, pH 3) were purchased locally (Ciudad Juarez, Chihuahua, Mexico; 31°44′22″N, 106°29′13″O), transported immediately under cooling conditions (2–4 °C), frozen (−80 °C), freeze-dried [−42 °C, 48 h; light-protected vessels (Labconco^TM^ Freezone 6, Labconco Co., Kansas City, MO, USA)], grounded to a fine powder (≤0.40 μm) and kept at −20 °C until use. Organic extracts (80% methanol) from all three freeze-dried samples (1:20 w/v; three batches per sample) were obtained by ultra-sonication (10 min; Fisher Scientific FS220H, Thermo Fisher Scientific, Waltham, MA, USA), centrifugation (4 °C, 15 min, 1650× *g*; Eppendorff^®^ centrifuge, mod. AG 5810R, Hamburg, Germany) and rotoevaporation (40 °C; Büchi^®^ R-114 evaporator, Büchi Labortechnik AG, Flawil, Switzerland). Freeze-dried samples were further dissolved in HPLC grade or Milli-Q water for analysis.

### 2.3. High-Performance Liquid Chromatography Quadrupole Time-of-Flight Mass Spectrometry (HPLC-ESI-QTOF-MS)

Identification of individual polyphenols in organic extracts (methanol 80%) was carried on an HPLC-ESI-QTOF-MS instrument according to Torres-Aguirre et al. [12]. Chromatographic separation was performed on an Agilent 1200 series system (Agilent Technologies, Palo Alto, CA, USA). The equipment was equipped with a vacuum degasser, an auto-sampler, and a quaternary pump. Extracts (sugar-free) were firstly separated using a reverse phase C_18_ analytical column (2.1 mm × 50 mm × 1.8 μm particle size; ZORBAX Eclipse Plus), protected with a guard cartridge of the same packing and maintained at 25 °C. The mobile phase [formic acid (0.1%) in Milli-Q water (A) and acetonitrile (B)] was pumped at 0.4 mL/min into the HPLC System. Two microliters were injected, and the sample was eluted following the gradient elution program: 0–4 min (90% A), 4–6 min (70% A), 6–8 min (62% A), 8–8.5min (40% A), 8.5–9.5 min (90% A) and the column was further re-equilibrated for 3 min. The quadrupole time-of-flight mass spectrometer (QTOF-MS; Agilent Technologies, Palo Alto, CA, USA) was coupled to a dual electrospray ionization (ESI) interface. The ESI-QTOF-MS operating conditions were: Source temperature (120 °C), gas desolvation temperature (340 °C), drying, nebulizing and collision gas (nitrogen; 13 L/min), capillary voltage (4.5 kV) and mass scan (100–1000 *m/z*). 

Before analysis, all samples (three batches x triplicate, *n* = 9) were filtered and concentrated by solid-phase extraction (Oasis HLB micro Elution plates, 96-well, 30 μm; Waters, Milford, MA, USA). Individual polyphenol identification was done by comparing the exact mass and molecular composition of the pseudo-molecular ion and/or quantification was performed by comparing with retention times (*rt*), UV-Vis spectra and molecular ion mass [*m/z* ± 0.1, M−H^+^ (anthocyanins and rutin) or M−H^−^ (all other polyphenols) mode] of pure phenolic standards (freshly prepared from stock solutions for each measurement), using the Mass Hunter Workstation Data Acquisition Software (ver. B.07.00; Agilent Technologies, Inc.) and an open-access MS-library (MassBank; https://massbank.eu/MassBank). The individual concentration of phenolic compounds was expressed in µg/g extract. Three different batches by triplicate (*n* = 9) from each fruit were evaluated.

### 2.4. Total Antioxidant Capacity 

Trolox equivalent antioxidant capacity (TEAC) of organic extracts (pure methanol; 1:20 w/v) per sample (three batches by quadruplicate, *n* = 12) was evaluated by the DPPH method (515 nm), the ferric ion reducing antioxidant power assay (FRAP, 630 nm) and the oxygen radical absorbance capacity [ORAC; fluorescein: 10 nM, (excitation (485 nm)/emission (520 nm), AAPH (240 mM)], as previously described [13], using a FLUOstar™ OMEGA spectrophotometer (BMG LABTECH; Chicago, IL, USA) in UV/VIS (DPPH, FRAP) and fluorescence (ORAC) modes. For all three assays a trolox standard curve [0.006–0.2 µmol/mL, R^2^ ≥ 0.95] was used. Values were expressed as mg or μmol of trolox equivalents (TE) per g (DPPH, FRAP) or µmole 1 × 10^10^ (ORAC) per g of freeze-dried sample ± standard deviation (*n* = 12) and as percentage considering blackberry antioxidant titers as 100% (sample with the highest total polyphenol content) [7]. 

### 2.5. In Vitro Digestion 

The method reported by Campos-Vega et al. [14] with minor modifications was used. For the oral stage, three otherwise healthy subjects were invited to participate in the study, providing written informed consent prior to participation. In fasting conditions and after brushing their teeth without toothpaste, each subject chewed each freeze-dried fruit (1 g × three batches, *n* = 3) 15 times for approximately 15 s. Chewed samples were collected into a beaker containing 5 mL of distilled water and subjects rinsed their mouths with another 5 mL of distilled water for 60 s. The volume of saliva + water was considered for data correction. For the gastric stage, pooled salivary samples per subject and sample were re-mixed per participant (*n* = 3) in an aseptic vessel and an aliquot (10 mL) was adjusted to pH 2 using HCl solution (2 N). Pepsin from porcine gastric mucosa (55 mg ≥ 250 units/mg protein, Sigma-Aldrich) dissolved in 0.94 mL of 20 mM hydrochloric acid was added to each sample and incubated for 2 h at 37 °C with constant agitation. For intestinal stage, a simulated intestinal extract was prepared 30 min before use by dissolving gall Ox (3 mg of bovine bile; CAS: 8008-63-7, Sigma-Aldrich) and porcine pancreatin (2.6 mg, 8 × USP, Sigma-Aldrich, St. Louis, MO, USA) in 5 mL Krebs–Ringer buffer (118 mM NaCl, 25 mM NaHCO_3_, 11 mM glucose, 4.7 mM KCl, 2.5 mM CaCl_2_, 1.2 mM MgSO_4_, 1.2 mM KH_2_PO_4_; pH 6.8]. Five mL of this solution were added to each sample coming from the gastric stage, pH adjusted to 7.2–7.4 with NaOH (2 M) and incubated for 2 h at 37 °C with constant agitation. All digested samples [three (pooled samples from gastric-to-intestinal phases per berry fruit) × triplicate, *n* = 9], were immediately transferred to the ex vivo bioanalytical system.

### 2.6. Rat Everted Gut Sacs 

Six small intestinal gut sacs were obtained from six young male albino rats (~300 g BW) which were fasted overnight (16–20 h) and anesthetized with an intraperitoneal injection of sodium pentobarbital (70 mg/kg BW, Pisabental, Guadalajara, Jalisco, Mexico), before surgical procedures and euthanasia, as suggested by Campos-Vega et al. [14]. Briefly, the intestine was exposed by a midline abdominal incision and 20–25 cm of the jejunal section was excised and placed in a gasified (CO_2_) Krebs-Ringer buffer solution at 37 °C. Each gut sac was gently washed externally with the same buffer and everted over a glass rod, re-excised into 6 cm-segments, filled (basolateral side) with 1 mL of Krebs-Ringer buffer (to avoid tissue denaturation) and fastened with braided silk sutures to a final length of approximately 4 cm [15]. Experiments were performed by triplicate and a blank was prepared using distilled water instead of in vitro digested sample. The experimental protocol was approved by the Animal Experimentation Ethics Committee of the Autonomous University of Ciudad Juarez (Code FO-CIP-01/254063; 30 July 2016) and animals cared according to the corresponding Mexican regulations (NOM-062-ZOO-1999) and the National Institutes of Health (NIH) guide for the care and use of laboratory animals. 

### 2.7. Real-Time Monitoring of Phenolic First-Pass Metabolism

**Figure 1:**
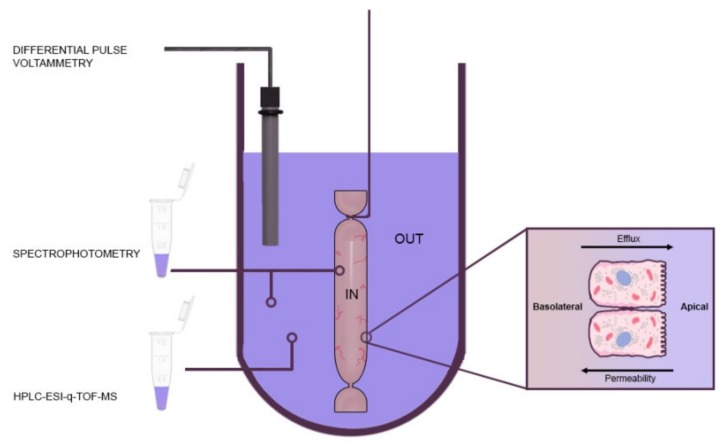
Real-time ex vivo monitoring of first-pass phenolic metabolism using the everted gut sac technique. The apparent permeability (P*app*) and biotransformation of bioaccesible phenolic compounds (PC) and their associated first-pass metabolites were followed by spectrophotometry (Folin–Ciocalteau; 120 min), HPLC-ESI-QTOF-MS (120 min) and, differential pulse voltammetry (DPV; 0 to 120 min). Diffusion from apical (A; out) to basolateral (B; in) and B-A were considered permeability and efflux, respectively.

The ex vivo first-pass metabolism biosystem (Figure 1) consisted of a 15 mL of pre-digested (oral-gastric-intestinal) samples (*n* = 3 per dried berry) and three 4 cm-closed everted duodenal sacs incubated in an oscillating (60–80 cycles/ min) water bath at 37 °C for 2 h, in an anaerobic chamber. Bioaccesible (from Section 2.6) and biotransformed polyphenols, withdrawn from the apical side (Figure 1, “out”), were tracked by three independent analytical methods: 

#### 2.7.1. Spectrophotometry (Method 1)

Total polyphenols were quantified spectrophotometrically (765 nm) with the FC method (TP_FC_) at the end of the experiment (120 min; *t_120_*); values were expressed as mean ± SD values [3 independent samples x triplicate, *n* = 9; mg of gallic acid equivalents (GAE)/ mL] as previously reported [7]. The apparent permeability coefficient (P_app_; Equation (1), efflux (ER; Equation (2) and uptake (UR; Equation (3)) ratios were calculated as follows, using the concentration of total polyphenols (Method 1) inside (basolateral; B) and outside (apical; A) the everted sacs at 120 min (*_t120_*):

P_app_ = (*ΔQ/Δt*) × (1−*AC_0_*)^−1^ (1)

ER= (B→A) × (A→B)^−1^ (2)

UR= (A→B) × (B→A)^−1^ (3)

where *ΔQ/Δt* is the steady-state flux (mg·s^−1^) of polyphenols transported across the membrane per second, *A* (cm^2^) is the surface area available for permeation and *C_0_* (mg/mL) represents the initial concentration of total polyphenols in the donor chamber (apical side of everted sacs; Figure 1). *P_app_* (mean ± SD) values were calculated and expressed in 10^−5^ cms^−1^.

#### 2.7.2. Differential Pulse Voltammetry (DPV; Method 2)

Differential pulse voltammetry (DPV) real-time (0–120 min) measurements of mixed polyphenols (parent + metabolites) were monitored (by triplicate) using a potentiostat (BASi^®^ EC Epsilon potentiostat/galvanostat; West Lafayette, IN, USA) and voltammetric measurements were carried out with a standard three-electrode electrochemical cell [working (glassy carbon, carefully polished with diamond spray, particle size 1 μM), counter (platinum wire) and reference (Ag|AgCl|KCl; 3M) electrodes]. Experimental conditions were: room temperature, pH, (7.2–7.4), scan range (0–600 mV) and rate (5 mVs^−1^), pulse width (70 ms) and amplitude (50 mV); these conditions were selected to avoid the interference of electrochemical species other than polyphenols and the current density (μA × 10^−5^) from the first (t_0_) oxidation peak (current *maxima*; mean = 203 mV, range 180 to 216 mV) was chosen as the reference value to estimate total polyphenols by DPV (TP_DPV_)

#### 2.7.3. HPLC-ESI-QTOF-MS (Method 3)

Non-targeted mass spectral identification (MassBank; https://massbank.eu/MassBank) and semi-quantification [as ion abundance (IA)] at *t_0_* and *t_120_* of parent polyphenols and their metabolites was performed by HPLC-ESI-QTOF-MS as reported above, following Koistinen et al. [10] recommendations. To avoid phytochemical loss by direct drying, individual samples (1 mL) were cleanup and concentrated by solid-phase extraction in Oasis HLB micro Elution 96-well plates (30 μm; Waters). Considering that real-time oxidation/reduction reactions readily occur within the ex vivo bioanalytical system used in this study, molecular ion identification was performed under the following considerations: *m/z* ± 0.3, M−H^+^ (anthocyanins and rutin) or M−H^−^ (all other polyphenols) mode].

### 2.8. Statistical Analysis

Results were expressed as mean ± standard deviation (SD) obtained from at least by triplicate. Inter-group (Red Globe grape, raspberry, blackberry) comparisons were performed by one-way-ANOVA followed by Tukey’s post hoc test and the statistical significance was defined at *p* < 0.05. When needed, Pearson’s product-moment correlation (*r*) was used to establish any possible correlation between response variables. Quadratic/cubic regression curves were constructed to explain electrochemical data (DPV). All statistics were performed using the statistical program NCSS 2007 (NCSS, Statistical Software, Kaysville, UT, USA).

## 3. Results and Discussion

### 3.1. Phenolic Profile of Berry Samples

Edible berries are rich in flavan-3-ols and anthocyanins that are barely affected during processing [16], although their content and molecular diversity is cultivar dependent [6]. In a preceding paper [7] we reported the spectrophotometric estimation (per g DW) of polyphenol subgroups in freeze-dried Red Globe grape, raspberry, and blackberry as follows: total polyphenols 9.4, 17.6 and 22.7 mg GAE, flavonoids 7.0, 13.1 and 35.3 mg quercetin equivalents (QE), monomeric anthocyanins (0.01, 0.49 and 0.67 mg cyanidin-3-*O*-glycoside equivalents), proanthocyanidins (0.22, 0.23 and 0.06 mg QE) and hydrolysable phenols (3.7, 7.2, 11.5 mg GAE). In the present study, we confirmed that these polyphenol subgroup titers correlate (*r* ≥ 0.76) with the overall content (ratio 0.07-0.14-1.0) and molecular diversity of flavan-3-ols and anthocyanins in the same fruits (blackberry> raspberry > Red Globe grape; Table 1). 

Our data also indicate that epicatechin, pelargonidin, and cyanidin-3,5-*O*-diglucoside (cyanin) were present in the evaluated samples, but catechin (Red Globe grape), kuromanin (cyanidin- 3-*O*-β-glucoside) and cyanidin-3-*O*-arabinoside (blackberry) and callistephin (pelargonidin-3-*O*- glycoside; raspberry and blackberry) were fruit-specific. 

Several anthocyanidins (aglycones) and related anthocyanins (3-*O*-glycosides and acyl glycosides) have been identified in grapes and shrubby berries. Colombo et al. [17] identified several flavan-3-ols (e.g., catechin, epicatechin, proanthocyanidin di/trimers), flavanols (quercetin and derivates), anthocyanins (all but pelargonidin glycosides), *cis*-resveratrol and caftaric acid (esterified phenolic acid) in Red Globe grape. However, pelargonidin (3,5,7,4’-tetrahydroxyflavium) and its 3-O-glycoside (callistephin) were not reported by these authors although they have been reported, in trace amounts, in certain grape varieties [18]. Also, blackberry and raspberry were better sources of polyphenols as compared to Red Globe grape, particularly in anthocyanin content. It is well-known that these berries are good sources of flavones (e.g., apigenin, chrysin), flavonols (e.g., kaempferol), phenolic acids (e.g., ellagic acid, caffeic acid), ellagitannins (e.g., sanguiin H-6, lambertianin C), anthocyanidins (all but peonidin) and anthocyanins such as cyanidin, delphinidin and pelargonidin glycosides [3,16,19].

The amount and natural occurrence of anthocyanidins (aglycone) and derived anthocyanins (glycosylated forms) are influenced by many factors including the type of cultivar and pre/postharvest handling of grapes [18], blackberries and raspberries [6]. Besides this, biosynthesis of anthocyanins in berry fruits is tightly controlled during the transcription of several genes involved in the flavan-3-ol proanthocyanidin pathway, in a fruit-specific manner [18,20]. Taking this into consideration, blackberry and raspberry are more valuable than Red Globe grape from a nutraceutical standpoint [1], even if their parent polyphenols are biotransformed into other ones [20,21]; the specific phenolic fingerprint of these two berries may be related to different yet complementary effects on preventing several non-communicable chronic diseases including certain types of cancers, cardiovascular disease, type II diabetes, inflammation and oxidative stress [2].

### 3.2. Antioxidant Capacity of Berry Samples

**Figure 2:**
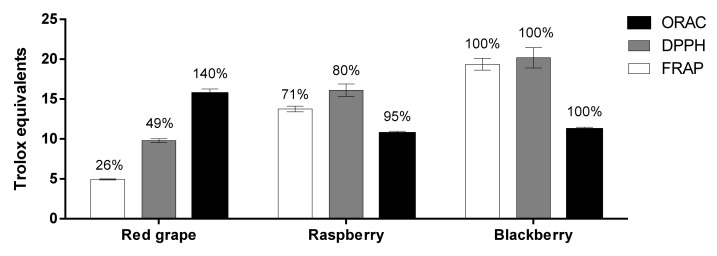
Antioxidant capacity of hydroalcoholic extracts from commercial *Red Globe* grape, raspberry and blackberry. Values were expressed as mean [*n* ≥ 9; mg TE/ gDW (DPPH, FRAP) or 1 × 10^1^ µmol TE/g DW (ORAC)]; percentages above bars indicate differences between samples considering blackberry antioxidant titers as 100%.

The antioxidant capacity of a given molecule (or a complex antioxidant mixture) is defined by its ability to reduce free reactive species (pro-oxidants or free radicals). The evaluation of the antioxidant capacity of plant-based foods by simultaneously using more than one method is a recommended practice in food science and technology [13].In this study, we used a single electron-transfer (SET; FRAP), a hydrogen atom transfer (HAT; ORAC) and a combined SET/HAT (DPPH) method to assay the antioxidant capacity in the studied samples (Figure 2). 

FRAP and DPPH values ranged between 5 (Red Globe grape)-19 (blackberry) and 10 (Red Globe grape)-20 (blackberry) mg TE/ g DW and from 11 (raspberry) to 16 (Red Globe grape) ×10^1^ µmol TE/ g DW with the ORAC method; similar trends in antioxidant capacity have been reported by other authors for the same berry fruits [3,16]. Also, the antioxidant capacity trend (blackberry > raspberry > Red Globe grape) was directly proportional to their phenolic content (Table 1), as measured by FRAP (100%-71%-26%; *r* = 0.86) and DPPH (100%-80%-49%; *r* = 0.84) methods but not with ORAC (100%-95%-140%; *r* = −0.42). DPPH and FRAP titers also correlate (*r* ≥ 0.95) with all polyphenol subgroups (total polyphenols, flavonoids, and anthocyanins) reported in our previous study [7] and same results have been reported for other polyphenol-rich fruits [6].

Conventionally, the higher the content of polyphenols in berry fruits, the higher their antioxidant capacity. It is important to point out that the radical scavenging capacity of most polyphenols is mediated by HAT rather than SET mechanisms. However, the antioxidant capacity is also related to the number and position of hydroxyl groups, the O–H bond dissociation enthalpy and conjugation/resonance effects [21]. Although the main antioxidant capacity mechanism in complex phytochemical mixtures is difficult to establish, the observed antioxidant capacity pattern (mostly blackberry > raspberry > Red Globe grape) apparently is the result of synergism between flavonoid species, the number of their available hydroxyl groups (particularly *O*-hydroxyls in A & B rings) and their level of glycosylation [21,22]. In support of this, Rice-Evans et al. [23] reported the following trend in antioxidant capacity with the ABTS radical (mixed SET/HAT mechanism): cyaniding > epicatechin/catechin > oenin > pelargonidin, and so, major drivers of the overall antioxidant capacity in the studied samples seem to be catechin (Red Globe grape), epicatechin (raspberry, blackberry) and cyanidin glycosides (blackberry). Lastly, cyanidin has a higher antioxidant capacity than its derived glycosides in the ORAC assay [24] and the catechin content shows a better lineal relationship with ORAC values than that observed with other flavan-3-ols [25]; whether these arguments justify Red Globe grape’s antioxidant activity in the ORAC method (Figure 2 merits future study.

### 3.3. Apparent Permeability of Berry Polyphenols

The net bioaccesibility (TP_FC_ = oral + gastric + intestinal) of polyphenols (as mg GAE per g DW) from Red Globe grape, raspberry, and blackberry was 2.0, 3.6 and 4.2 (A*_t0_*, Table 2) which represents 21.3, 20.4 and 18.6% of their original content [7]. 

In our preceding study we also reported that anthocyanins, but no other flavonoids were pH-unstable under simulated intestinal conditions (pH 7.0); similar results have been reported for strawberry [26], maqui berry [27] and blueberry [28]. After intestinal digestion (Table 2), 1.3, 1.5 and 2.4 mg GAE per g DW remain in the apical side (A_t120_) suggesting 35%, 58% and, 43% of net polyphenol absorption; however, the basolateral (serosal) concentration of polyphenols at 2h (B_t120_) was blackberry (0.13) > Red Globe grape/ raspberry (~0.095) and the absorptive (A_t120_→B_t120_) and secretory (B_t120_→A_t120_) P_app_ (cm·s^−1^ × 10^−5^) were Red Globe grape (1.20)> blackberry (0.06)/ raspberry (0.07) and Red Globe grape (1.55)/blackberry (1.38) > raspberry (0.98), respectively. In consequence, efflux (19.1, 16.1, 1.3) were higher than uptake (0.05, 0.06, 0.78) ratios were fruit-specific (*p* ≤ 0.02) and concentration-dependent (blackberry > raspberry > Red Globe grape). 

Many transport mechanisms seem to be involved in the uptake/efflux behavior of polyphenols. Dixit et al. [29] used a standardized everted sac-based biosystem to evaluate the permeability behavior of atenolol (Pubchem CID: 2249; XLogP3 = 0.2, simple paracellular transport), metoprolol (Pubchem CID: 4171; XLogP3 = 1.9, transcellular transport) and propranolol (Pubchem CID: 4946; XLogP3 = 3.0; passive diffusion), reporting absorptive (A→B) *P_app_* values of 0.054, 0.84 and 1.64 cms^−1^ × 10^−5^, respectively. Considering miLogP values reported in Table 1, and the fact that anthocyanins and anthocyanidins cannot cross cell membranes passively, passive (Red Globe grape) and paracellular transport (blackberry/raspberry) may be major transport mechanisms in the studied samples. 

Molecular bioinformatics provided information on the capability of each berry polyphenol to be absorbed by the intestinal epithelia. Most polyphenols listed in Table 1 had a topological polar surface area [TPSA; 92.1 (pelargonidin) to 270.6 (cyanin)] and octanol/ water partition coefficient [LogP; −4.61 (cyanin) to 1.37 (catechin/epicatechin)]. It is known that phytochemicals with a TPSA > 140 Å^2^ or ≤ 60 Å^2^ have low and high permeability, respectively and those neutral or with a LogP > 2.0 easily permeate by passive diffusion [30]. The “pH partition” hypothesis postulates that non-ionized (neutral) molecules are passively transported through the lipid membranes [30] and according to the modified *theoretical transcellular permeability* model proposed by Farrell et al. [31], the *P_app_* of monomeric polyphenols is well explained by *logP* and molecular weight. Based on this, active more than passive transport and regular permeability of polyphenols from the assayed berries should be expected [8,9].

However, P-glycoprotein (*P-gp* or MDR-1) and breast cancer resistance protein (BCRP) can simultaneously reduce the first-pass bioavailability of certain polyphenols by acting as efflux modulators [5,32], as it has been proposed for polyphenols coming from spent coffee [14] and mango bagasse [33]. In fact, certain polyphenols may also act as competitive inhibitors of *P-gp* since it has a high affinity toward molecules with a planar ring system ranging from 200 to 1900 Da [32]. However, uptake/efflux behavior is also an asymmetric function that depends on the chemical structure of a particular polyphenol to be absorbed and the rate to which it is bioaccesible during *in vivo* or *in vitro* gastrointestinal conditions [14,33,34]; but such dynamic changes in transport behavior could not be observed with our endpoint assay (Method 1). 

### 3.4. Ex Vivo Biotransformation of Berry Polyphenols 

As previously discussed, several in vitro and ex vivo permeability models have been developed to mimic specific aspects of gastrointestinal metabolism, each one with advantages and disadvantages [15,29]. Particularly, the sensitivity and reliability of the everted gut sac technique increase when combined with high-throughput detection methods such as electrochemical and HPLC/MS-based methods [10,34]. On the other hand, experimental data such as retention time (*rt*), mass-spectra (*m/z*^+*/−*^) of a particular standard molecule is often used in targeted metabolomics (Level 1) to objectively identify and quantify the same molecule in a given biological sample that presumably contains it. Conversely, untargeted metabolomics (Level 2) focuses on the global detection and relative quantitation of metabolites of unknown chemical nature, and their putative identification and semi-quantification rely upon spectral matching with databases (e.g., MassBank) or as suggested in earlier publications [10]. In this study, a level 2 strategy was used to partially identify (HPLC-MS-QTOF-MS) and semi-quantify (DPV current *maxima* and ion counts) of major [effluxed (B-A)] metabolites coming from the *ex vivo* biotransformation of parent polyphenols (Table 1). 

#### 3.4.1. DPV

**Figure 3:**
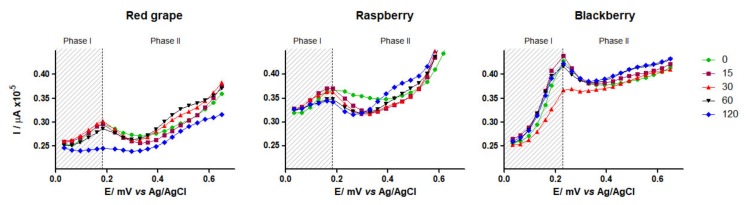
Differential pulse voltammograms of post-digested berry samples, during their *ex vivo* apparent permeability and biotransformation. Polarization rate 5mV*s^−1^ (abscissa values × 10^−3^); phase I (“absorption”; grey rectangle), phase II (“biotransformation” open white), pH = 7.2–7.4.

Voltammetric methods are useful to predict the antioxidant activity of plant foods [11] and biological samples. Particularly, *maxima* DPV anodic current highly correlates with the antioxidant capacity (FRAP, ABTS and, DPPH) and concentration (HPLC and spectrophotometry) of pure polyphenols [35,36] but it is more sensitive (μM vs. mM) to quantify total polyphenols (TP_DPV_) in grapes, raspberries and red wines [11,19]. DPV has been also used to evaluate the *P_app_* of specific drugs in vitro using intestinal cell monolayers or ex vivo using intestinal reperfusion models. However, to our knowledge, reports on the electrodynamics of polyphenols during their first-pass metabolism using an everted gut sac have not been reported before. DPV voltammograms (vs. Ag|AgCl|KCl reference electrode) of pre-digested berries at neutral pH (Figure 3) showed the following trend [potential peak (mV)/ anodic current density (μA × 10^−5^)]: 180/0.24 (*Red Globe* grape) > 216/0.34 (raspberry) > 212/0.44 (blackberry); the estimated TP_DPV_ ratio was 0.6:0.8:1.0 which linearly correlated (r ≥ 0.95) with TP_FC_ (Table 2) and antioxidant capacity (Figure 2; DPPH and FRAP). 

The oxidation potential of monomeric polyphenols depends on the amount and position (*ortho* or *para* > *meta*) of reactive hydroxyl groups in benzene ring(s) and the *ortho*-effect between two hydroxyl groups or hydroxyl/carbonyl groups [35]. Alcalde et al. [36] evaluated the relationship between the molecular structure and electrochemical behavior of fifteen polyphenols by DPV, demonstrating that polyphenols with higher sensitivities (lower DPV potential) are strong antioxidants and that flavonoids are more electroactive (lower potentials) than phenolic acids (higher potentials); they also found that catechin exhibits two oxidation peaks at ~200 (A ring) and ~600 (B ring) mV (vs. Ag|AgCl|KCl reference electrode) at pH 5.0, while at pH 7.5 [37] and 3.6 [11] it exhibits both oxidation peaks at 148/537 mV and ~450/750 mV, respectively.

Kuromanin and cyanin are major anthocyanins in berry fruits [3,16] both exhibiting the same oxidation potential in their catechol group [P1 (current peak): B-ring; SET mechanism, reversible reduced] but different oxidation behavior at their resorcinol moiety (P2: A-ring, SET mechanism, irreversible) associated with the additional glucosylation (shift right: +95 (pH 3.5–4.5), +130 (pH 7.0) mV] and P1 shifts to lower potentials (vs. Ag|AgCl|KCl reference electrode) when increasing pH [20,21,22]. 

The aforementioned inverse relationship between pH and oxidation potential has been also demonstrated for several anthocyanins from *Vitis vinifera* [22]. Together, this evidence supports the idea that the observed DPV current peak *maxima* at *t_0_* in all three voltammograms (Figure 3) may be partially explained by a berry-specific amount of polyprotic species at neutral pH (7.2–7.4), which is the case of the not-methylated anthocyanidins and anthocyanins reported in Table 1. It is worth mentioning that ellagic acid, the most representative phenolic acid in berries, exhibits a very low oxidation potential nearby neutral pH [38] and so, it seems that it does not contribute to the observed current peak *maxima* at *t_0_* in all three samples (Figure 3).

Since redox processes and apparent permeability of polyphenols were concurrent events in the *ex vivo* bioanalytical system used here, we used time-trend kinetic data to gain more insights on such events. For this purpose, the following procedure was followed: *A)* The real-time electrodynamic behavior exhibited by each pre-digested sample (Figure 3) from 0 (basal) to 120 min was arbitrarily divided into two segments, before and after current *maxima* [Red Globe grape (180 mV), raspberry (216 mV), blackberry (212 mV)], and labeled as “absorption" (stage 1) and "metabolite production" (stages 2); here we postulate that a time-trend reduction of current density before *maxima* current peak is mainly due to an apparent permeability and/or structural modification of parental polyphenols while increments after peak *maxima* are more likely to be due to new chemicals species with reduced electroactivity. *B)* Goodness-of-fit regression models for stage 1 (quadratic) and stage 2 (cubic) were then obtained and, *C)* theoretical current density values (TCD; μA × 10^−5^) were calculated using the mean potential peak value (203 mV) at each time point (Table S1 and Figure S1). 

During stage 1, a time-trend lineal reduction of TCD for Red Globe grape (R^2^ = 0.91) but not for raspberry or blackberry was documented (Table S1 and Figure S1); this seems to be related to the lower amount and less diverse polyphenol profile (high catechin) in this table grape (Table 1), to higher electrochemical stability and apparent permeability and to a lower efflux of polyphenols (Table 2) than that observed for raspberry and blackberry. It is known, that anthocyanins>flavan-3-ols are pH-sensitive under simulated intestinal conditions [7,28], that anthocyanidins are more electroactive than their corresponding anthocyanins [24], that anthocyanin di-glycosides are more stable than mono-glycosides [21,22] and that GLUT-2 and SGLT-1 are efficient transporters for anthocyanins and flavan-3-ols at lower apical concentrations while *P-gp*/MCRP efflux systems are activated upon GLUT-2/SGLT-1 saturation [33,39,40]. 

During stage 2, less electroactive (~400–600 mV) species were erratically produced (X^3^-behavior) in a berry-specific trend (Figure 3, Table S1 and Figure S1): raspberry (X^3^ range = 3 to 10)> Red Globe grape (X^3^ range= −2 to 0.2)> blackberry (X^3^ range= −2 to −9). DPV is also useful to study the real-time redox phenomena in vitro (particularly under acidic conditions) such as the time-course photo-degradation of 4-acetamidophenol (a.k.a. acetaminophen) [41] or the electro-Fenton degradation of 3-methyl phenol (*m*-cresol) [42] in which new molecules with higher (acetaminophen) or lower (*m*-cresol) potentials are produced from these synthetic phenolics. 

However, to our knowledge, the real-time degradation of natural polyphenols under neutral pH has not been reported yet, nor the use of DPV for monitoring their time-trend enteral biotransformation. Although the evidence points out to a berry-specific ex vivo biotransformation of parental polyphenols, the chemical nature of phenolic metabolites could not be evidenced by this method. Nonetheless, partially oxidized flavan-3-ols and anthocyanins > phenolic acids seem to be predominant antioxidant species at neutral pH within the narrowed potential range used in this study (0−600 mV), a fact previously reported by other authors [35,37]. 

#### 3.4.2. HPLC-ESI-QTOF-MS

The European Cooperation in Science and Technology Commission (COST; FA-1403 POSITIVe action) recommends the use of high through-output analytical platforms in untargeted metabolomics to evaluate the inter-individual variability in the physiological response to phytochemical intakes [4,10]. Particularly, HPLC-ESI-MS^n^ is widely used in untargeted polyphenol metabolomics [19,28]; such a platform was used here to track the ex vivo small gut biotransformation (2 h; end-point assay) of parent polyphenols from three berries with graded levels of phenolic compounds [7]. Table 3 shows the chemical nature and apparent content of bio-accessible polyphenols (released by in vitro digestion) before their ex vivo biotransformation (t_0_) that substantially differed from those identified in the assayed fruits who were chemically extracted (Table 1).

**Figure 4:**
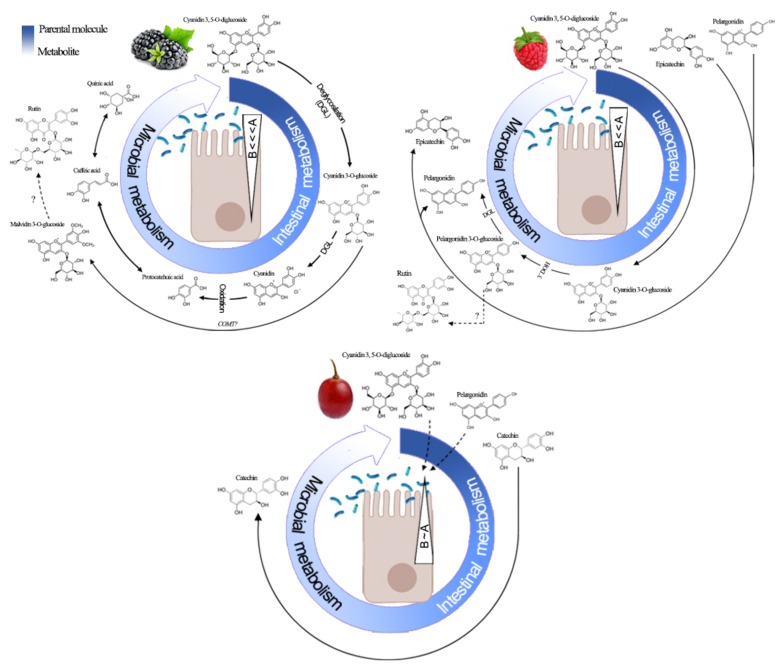
First-pass metabolism of polyphenols from blackberry, raspberry and *Red Globe* grape. Stepwise metabolite production from parental polyphenols (Table 1) detected by HPLC-ESI-QTOF-MS (both, below (Table 3) and over (Table S2) a signal-to-noise 10:1 ratio results from a concerted action of epithelial enzyme machinery and jejunal microflora. Triangle magnitude and direction (A<~>B) is derived from Table 2. Apical (A), basolateral (B), catechol-*O*-methyltransferase (COMT), 3’ hydroxyl removal (3’DOH), deglycosylation (DGL).

Such a difference is even more evident when considering the HPLC-ESI-QTOF-MS profile at a signal-to-noise ratio < 10:1 (Table S2; values at *t_0_*). The same has been reported for black and green currants [43] and strawberries [26] when comparing the polyphenolic profile of these berries before and after *in vitro* digestion, it was observed that not only the quantity of parental polyphenols but also their chemical nature differed (also observed in Figure 4). Many biological and analytical factors are involved in this phenomenon, including the pH-instability (neutral > acidic) of polyphenols, particularly anthocyanins [8,20], the REDOX status of parent/metabolites (*m/z* ± 0.3), HPLC-ESI-QTOF-MS limit of detection (10:1 signal-to-noise) and their reversible interaction with digestive enzymes and mucin [5,10,44]. 

Steinert et al. [45] using a CaCO_2_ monolayer transport system showed that the apical-to-intracellular transport of blackcurrant anthocyanins occur faster than their translocation across the basolateral membrane and that ~11% of all anthocyanins disappeared from the apical chamber within the first twenty minutes; the authors concluded that cell metabolism rather than apparent permeability was involved in the first-pass metabolism of black currant anthocyanin. Kuntz et al. [39] also studied the apparent and apical bioavailability of anthocyanins from grape/blueberry juice and smoothie permeability in transwell chambers with and without Caco-2 cell (ATCC^©^ HTB37^TM^) monolayers, showing that both specific and absolute anthocyanin concentration decreased overtime in apical chambers without cells at neutral (7.4) but not acidic (2.0) pH and that total anthocyanin disappearance were even more evident with cells than without them. Both research groups also documented a structure-specific disappearance rate of anthocyanins due to concurrent absorption and biotransformation processes. 

Another plausible explanation comes from microbial biotransformation. As previously mentioned, the absorptive behavior [P_app_ (A*_t120_*→B*_t120_*)] and uptake ratio [(A→B) × (B→A)^−1^] was inversely related to the fruit-specific polyphenol-richness and luminal biotransformation (Figure 4). This implies that the resident time of parent polyphenols, particularly those from blackberry and raspberry, in the apical side was long enough to be used as substrates for brush border enzymes and possibly by the resident microbiota including but not restricted to *Lactobacillus* sp., *Actinobacterium* sp. and *Clostridium* sp. which together represents ~70% of normal rat duodenal microbiota [46] and whose substrate preference include flavonoids, anthocyanins, and ellagitannins [47]. However, depending on the composition of the microbiota, different metabolites may be produced from the microbial biotransformation (postbiotics) of berry anthocyanins, despite the fact that certain phenolic acids and flavonoids may also act as prebiotics [2]; this double effect of polyphenols enlarges their recognized health benefits [1,3,4,5].

The 2 h (*t_0_* vs*. t_120_*) ex vivo exposure to the intestinal epithelium reflected both the epithelial in/out interchange discussed above (see dotted triangles in Figure 4) and a great biotransformation phenomenon characterized by a low or no detection of parental anthocyanins and anthocyanidins and higher production of small molecular weight (≤ 354 gmol^−1^) polyphenols (Table 3, Table S2). Extensive and rapid deglycosylation of anthocyanins occurs in vivo and ex vivo releasing anthocyanidins with a reduced polarity (less TPSA). The resulting anthocyanidins may be either absorbed by passive (paracellular) diffusion or subject to microbial breakdown (particularly on B ring) producing phenolic acids (e.g., protocatechuic, chlorogenic and caffeic acids) and polyols (e.g., quinic acid; Figure 4) [2,9] and C_6_-C_3_-C_6_-derived intermediates [44]. Chen et al. [8] followed the in vitro bioaccessibility and biotransformation of kuromanin under simulated GI conditions showing that this anthocyanin rapidly disappears but a wide range of metabolites (namely protocatechuic acid and derivates, cyanidin, caffeic and ferulic acids) were produced instead, all of them showing different permeability behaviors. 

Lastly, since cyanin just have and additional glucose moiety at 5’ when compared to kuromanin, its metabolic fate may be the same after enzymatic deglycosylation [9]. Kuromanin (and possibly cyanin) is partially deglycosylated by β-glucosidase (EC 3.2.1.21) and lactase-phlorizin hydrolase (EC 3.2.1.108) but it undergoes extensive *in vivo* biotransformation to low molecular weight breakdown metabolites and, a wide range of phase II metabolites including anthocyanin methylation [20]; since some of these metabolites are either reported in Table 3 or Table S2, this partially supports an extensive kuromanin biotransformation from blackberry and raspberry (Figure 4). Although a straightforward identification of C_6_-C_3_-C_6_ compounds derived from kuromanin, cyanin or callistephin has not been reported yet, the removal of functional groups *ex vivo* (as hypothesized in this study) may interconvert anthocyanidins (e.g., the loss of a hydroxyl group from the B-ring of cyanidin gives rise to pelargonidin) as it has been shown in vivo [44]. The biotransformation of chlorogenic (Table 3) and protocatechuic (Table S2) acids, two of the most abundant phenolic acids in edible fruits, gives quinic and caffeic acid (Figure 4) whose further methylation gives ferulic and isoferulic acids [48]; however, whether the intestinal or microbial catechol-*O*-methyltransferase (COMT; EC 2.1.1.6) activity is involved in the transformation of kuromanin into callistephin (from blackberry), deserves further study.

## 4. Conclusions

A moderate permeability (~20%) and a high ex vivo biotransformation of parent polyphenols (molecular breakdown and isomerized anthocyanin products) from the assayed berries were found in this study. This partially suggests fruit-specific health effects, most likely at the intestinal level due to a higher efflux phenomenon. The ex vivo high-through output bioanalytical approached used here [everted gut sac (rat) + three detection methods: spectrophotometry, HPLC-ESI-QTOF-MS, differential pulse voltammetry (DPV)], provided important information on berry polyphenol biotransformation during their ex vivo first-pass metabolism that may help to understand the metabolic fate and effects of the studied berries; however, further studies are needed to understand the biological activities of biotransformed phenolics and not only their parental molecules.

## Figures and Tables

**Table 1:** HPLC-ESI-q-TOF-MS and cheminformatics of polyphenols from three edible berries ^1,2^.

Compound	*rt*	*m/z*	Grape	Raspberry	Blackberry	TPSA	LogP
Catechin	1.5	289.1	86 ± 10	--	--	110	1.37
Epicatechin	2.7	289.1	10 ± 5 ^c^	451 ± 6 ^b^	1121 ± 95 ^a^	110	1.37
Cyanidin-3-*O*-β-glucoside	4.2	450.1	--	--	2762 ± 31	181	0.34
Cyanidin-3-*O*-arabinoside	5.6	420.2	--	--	21 ± 0	161	−2.37
Pelargonidin	6.6	272.1	67 ± 4 ^a^	47 ± 1 ^b^	65 ± 2 ^a^	82	−0.26
Pelargonidin-3-*O*-glucoside	7.1	433.2	--	20 ± 3 ^a^	15 ± 0 ^b^	171.2	−2.30
Cyanidin-3,5-*O*-diglucoside	7.9	612.4	134 ± 8 ^a^	58 ± 4 ^c^	111 ± 3 ^b^	270.6	−4.61
Total polyphenols			297 ± 27 ^c^	576 ± 14 ^b^	4095 ± 131 ^a^		

^1^ Results are expressed as mean (*n* ≥ 9) ± standard deviation (µg /g dry weight basis); different superscript letters between samples for a same compound means statistical differences (*p* < 0.05); retention time (*rt*, min), molecular ion [*m/z* ± 0.3, positive (anthocyanins) or negative (flavan-3-ols) mode], below quantification limit (--). ^2^ Total polar surface area (TPSA. Å^2^) and octanol/water partition coefficient (LogP) values were retrieved from Molinspiration chemoinformatics (https://www.molinspiration.com/), using each compound’s canonical SMILE sequence retrieved from PubChem (https://pubchem.ncbi.nlm.nih.gov/.

**Table 2:** Apparent permeability of phenolic compounds from selected berries.^1,2.^

Parameter	Red Globe Grape	Raspberry	Blackberry
A*_t0_* (TP_FC_)	2 ± 0.0 ^c^	3.6 ± 0.1 ^b^	4.2 ± 0.1 ^a^
A*_t120_*	1.3 ± 0.3 ^b^	1.5 ± 0.1 ^b^	2.4 ± 0.1 ^a^
B*_t120_*	0.10 ± 0.0 ^b^	0.09 ± 0.0 ^b^	0.13 ± 0.0 ^a^
Absorptive P_app_ (A*_t120_*→B*_t120_*)	1.20	0.06	0.07
Secretory P_app_ (B*_t120_*→A*_t120_*)	1.55	0.98	1.38
Efflux ratio (B→A)*(A→B)^−1^	1.29	16.12	19.12
Uptake ratio (A→B)*(B→A)^−1^	0.78	0.06	0.05
*p* (ER vs. UR)	0.02	0.002	<0.0001

^1^ Results are expressed as mean ± standard deviation (*n* ≥ 9; mg GAE /g dry weight; Folin-Ciocalteu method), different superscript letters within a same row means statistical differences (*p* < 0.05). ^2^ Total polyphenol content by the Folin-Ciocalteu method (TP_FC_), basal (*t_0_*) and final (*t_120_*) apical (A) or basolateral (B) concentration. Apparent permeability coefficient (P_app_; cm*s^-1^ x 10^-5^). Statistical difference between efflux (ER) vs. uptake (UR) ratios as determined by t-student test (*p* < 0.05).

**Table 3:** First-pass metabolism of phenolic compounds from selected berries: HPLC-ESI-q-TOF-MS ^1,2^.

Sample	Phenolic	*rt*	*m/z*	Ion Abundance (IA)		Δ (%)
				t_0_	t_120_	
Raspberry	Quinic acid	0.6	191.1	104,000 ± 1061	45,600 ± 636	−56 ± 0
	Epicatechin	3.0	289.1	9500 ± 707	3100 ± 141	−67 ± 1
	Cy3G	4.2	450.1	16,500 ± 707	4750 ± 354	−71 ± 1
Blackberry	Quinic acid	0.6	191.0	8950 ± 212	4600 ± 566	−47 ± 9
	Chlorogenic acid	1.6	353.1	71,300 ± 1768	47,300 ± 354	−34 ± 1
	Caffeic acid	2.3	179.0	3750 ± 354	23,500 ± 707	530 ± 78
	Ma3G	4.9	494.1	950 ± 71	8450 ± 354	1006 ± 8

^1^ Results are expressed as mean (*n* ≥ 9; ion counts) ± standard deviation of selected phenolic compounds detected with a signal-to-noise ratio ≥10:1. ^2^ Retention time (*rt*, min), mass-to-charge ratio [*m/z* ± 0.1, positive (anthocyanins) or negative (all other polyphenol) ion mode], cyanidin (Cy3G; kuromanin) or malvidin (Ma3G; oenin)-3-*O*-glucosides; initial (A*t_0_*), final (A*t_120_*) and change (Δ (%) = [1-( *t_120_/ t_0_*)] × 100), apical ion abundance; reduced (−), increased (+).

## Supplementary Materials

The following are available online at https://www.mdpi.com/2076-3921/9/4/311/s1, Table S1 and Figure S1: Voltamperometric behavior of mixed antioxidants from three berry fruits, Table S2: First-pass metabolism of polyphenols from selected fruits- HPLC-ESI-q-TOF-MS (signal-to-noise ratio < 10:1).
