# Supplementary material for: First-Pass Metabolism of Polyphenols from Selected Berries: A High-Throughput Bioanalytical Approach
Source: Antioxidants (Basel). 2020 Apr 13;9(4):311. doi: 10.3390/antiox9040311 (PMC7222205; doi:10.3390/antiox9040311)
Supplement: Supplementary file 1 [file antioxidants-09-00311-s001.pdf]

Table S1. Voltamperometric behavior of mixed antioxidants from three berry fruits.

Fruit	Time	Phase 1 "absorption"					Phase 2 "metabolite production"				
	min	X^2	X	b	R^2	TCD	X^3	X^2	X	b	R^2
Grape	0	2.00	-0.01	0.20	0.98	0.28	0.10	1.00	-0.70	0.30	0.99
	15	0.90	0.09	0.20	0.99	0.25	0.20	1.00	-1.00	0.40	0.99
	30	0.80	0.20	0.20	0.99	0.27	-2.00	3.00	-2.00	0.40	0.98
	60	1.00	-0.05	0.20	0.99	0.23	-3.00	5.00	-2.00	0.50	0.98
	120	0.80	-0.20	0.20	0.95	0.19	-2.00	3.00	-1.00	0.30	0.99
Raspberry	0	2.00	0.05	0.30	0.98	0.39	9.00	-9.00	3.00	0.10	0.99
	15	-0.04	0.50	0.30	0.98	0.40	10.00	-10.00	2.00	0.20	0.99
	30	-0.80	0.50	0.30	0.98	0.37	6.00	-3.00	0.10	0.40	0.96
	60	-0.20	0.30	0.30	0.97	0.35	10.00	-9.00	2.00	0.09	0.98
	120	1.00	0.40	0.30	0.97	0.42	3.00	-0.70	-0.20	0.30	0.95
Blackberry	0	7.00	-0.50	0.20	0.99	0.38	-5.00	8.00	-4.00	1.00	0.97
	15	5.00	0.01	0.20	0.98	0.40	-9.00	10.00	-6.00	1.00	0.94
	30	4.00	-0.20	0.20	0.99	0.32	-2.00	4.00	-2.00	0.50	0.99
	60	4.00	0.20	0.20	0.97	0.40	-7.00	10.00	-5.00	1.00	0.97
	120	5.00	0.20	0.20	0.98	0.44	-7.00	10.00	-5.00	1.00	0.97

Theoretical current density (TCD; $\mu A \times 10^{-5}$) at peak current *maxima* (mean value= 0.2 V)

Figure S1. Voltamperometric behavior of mixed antioxidants from three berry fruits.

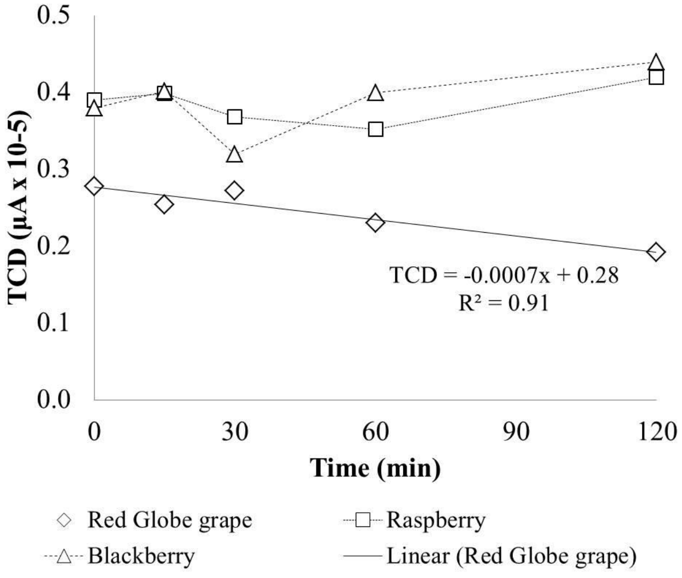

Table S2. First-pass metabolism of polyphenols from selected fruits- HPLC-ESI-q-TOF-MS (signal-to-noise ratio <10:1) [1,2]

Sample	Tentative PC	*RT*	*m/z*	Ion abundance (IA)		Δ (%)
				t_0	t_{120}	
Red Globe grape	Catechin	1.8	289.1	348 ± 32	222 ± 5	-36 ± 7
Raspberry	Gallic acid	0.7	169.1	525 ± 35	288 ± 53	-45 ± 14
	Protocatechuic acid	1.0	153.1	375 ± 106	273 ± 4	-24 ± 23
	Chlorogenic acid	1.6	353.1	400 ± 141	--	-100
	Catechin	1.8	289.1	713 ± 18	345 ± 21	-52 ± 4
	Caffeic acid	2.3	179.1	--	235 ± 21	+100
	Ellagic acid	3.1	301.1	2,380 ± 177	1180 ± 106	-51 ± 1
	Rutin	4.1	611.1	235 ± 21	275 ± 35	+18 ± 26
	Ma3G	4.9	493.1	1250 ± 71	--	-100
	Petunidin	6.1	318.1	2,400 ± 141	613 ± 17	-74 ± 2
Blackberry	Gallic acid	0.7	169.1	BQL	360 ±28	+100
	Protocatechuic acid	1.0	153.1	413 ± 18	275 ± 35	-33 ± 11
	Catechin	1.8	289.1	BQL	610 ± 14	+100
	Rutin	4.1	609.1	2100 ± 141	2750 ± 71	+31 ± 6
	Cy3G	4.2	449.1	605 ± 64	1,400 ± 141	+132 ± 1
	Pelargonidin	6.5	271.1	825 ± 35	BQL	-100

[1]Results are expressed as mean ion abundance (counts) ± standard deviation of phenolic compounds tentatively identified detected with a signal-to-noise ratio <10:1. [2] Retention time (*RT*, min), mass-to-charge ratio [*m/z* ± 0.3, positive (anthocyanins, rutin) or negative (all other polyphenol) ion mode], initial (At_0), final (At_{120}) and change (Δ (%) = [1-(t_{120}/ t_0)]*100), apical ion abundance; reduced (-), increased (+); Cyanidin (Cy3G; chrysanthemin) or malvidin (Ma3G; oenin)-3-*O*-glucosides.
